# Frequency-Specific Fractal Analysis of Postural Control Accounts for Control Strategies

**DOI:** 10.3389/fphys.2018.00293

**Published:** 2018-03-28

**Authors:** Pierre Gilfriche, Véronique Deschodt-Arsac, Estelle Blons, Laurent M. Arsac

**Affiliations:** ^1^CATIE - Centre Aquitain des Technologies de l'Information et Electroniques, Talence, France; ^2^Univ. Bordeaux, CNRS, Laboratoire IMS, UMR 5218, Talence, France

**Keywords:** postural control, center-of-pressure, fractal physiology, DFA, FsFA, 1/f scaling

## Abstract

Diverse indicators of postural control in Humans have been explored for decades, mostly based on the trajectory of the center-of-pressure. Classical approaches focus on variability, based on the notion that if a posture is too variable, the subject is not stable. Going deeper, an improved understanding of underlying physiology has been gained from studying variability in different frequency ranges, pointing to specific short-loops (proprioception), and long-loops (visuo-vestibular) in neural control. More recently, fractal analyses have proliferated and become useful additional metrics of postural control. They allowed identifying two scaling phenomena, respectively in short and long timescales. Here, we show that one of the most widely used methods for fractal analysis, Detrended Fluctuation Analysis, could be enhanced to account for scalings on specific frequency ranges. By computing and filtering a bank of synthetic fractal signals, we established how scaling analysis can be focused on specific frequency components. We called the obtained method Frequency-specific Fractal Analysis (FsFA) and used it to associate the two scaling phenomena of postural control to proprioceptive-based control loop and visuo-vestibular based control loop. After that, convincing arguments of method validity came from an application on the study of unaltered vs. altered postural control in athletes. Overall, the analysis suggests that at least two timescales contribute to postural control: a velocity-based control in short timescales relying on proprioceptive sensors, and a position-based control in longer timescales with visuo-vestibular sensors, which is a brand-new vision of postural control. Frequency-specific scaling exponents are promising markers of control strategies in Humans.

## Introduction

The regulation of human posture is operated via several feedback loops compensating internal and external disturbances (Diener et al., 1984). Postural sway in the upright position is the oscillation of the body maintaining its static balance in the orthostatic position. Study of the postural sway has shown to be a valuable indicator to assess the quality and characteristics of the regulation of posture. It was used among other applications for assessment of frailty in elderly people (Jiang et al., 2013; Zhou et al., 2017), to predict falls in chronic stroke survivors (Punt et al., 2017), in the identification of idiopathic scoliosis (Gruber et al., 2011), in differentiating postural strategies in athletes and non-athletes (Paillard et al., 2002; Lamoth and van Heuvelen, 2012), to analyze the effects of a dual task (Kang et al., 2009), or of fear of falling (Davis et al., 2009) on posture. Most importantly, it has become an indicator of efficiency for rehabilitation procedures (Priplata et al., 2002; Costa et al., 2005; Chen and Jiang, 2014; Wayne et al., 2014).

Postural sway is generally studied through recordings of the time course of the center-of-pressure (CoP) of a subject on a static platform. CoP is the point of application of the resultant of vertical forces acting on the surface of support, and is derived from ground reactions. The stabilogram is the trajectory of this point in the two-dimensional plane (CoP signal). Most analysis are based on time-series of the projection of the stabilogram along the antero-posterior (AP) and medio-lateral (ML) axis, as a consequence of the human body's symmetry (Błaszczyk and Klonowski, 2001). From a practical perspective, projection allows classical methods for the analysis of one-dimensional time-series to be used.

Several indicators have been designed over the years to quantify postural control and upright stability. Basic ones consist in quantifying the variability in the CoP signal. Great variations have often been associated with weak control (Horak et al., 1989) since they are associated with bigger average distance to the equilibrium and higher energy expenditure. Numerous metrics used to characterize the CoP are regularly used in the literature (Jiang et al., 2013; Paillard and Noé, 2015): sway length, standard-deviation along the AP and ML direction, CoP mean velocity, 90 or 95% confidence elliptic area, to name a few.

Early in the 70s, the frequency content of the projections of CoP signal has been a matter of interest. Particular cerebellar lesions or proprioceptive hyperactivity were associated to alterations in specific frequency in the power spectrum of postural sway (Taguchi, 1977). Typical indicators derived from frequency analysis are the absolute or relative power in the identified frequency bands. It has been reported in pioneer works that frequencies between 2 and 20 Hz, so-called high frequencies in CoP signal analyses, mostly account for somatosensory feedback mechanisms (Dietz et al., 1980; Golomer et al., 1994). The main sensors involved in short spinal loops associated to these high frequencies are likely muscle spindles, Golgi tendon organ, and cutaneous receptors (Aniss et al., 1990). Other authors have considered the frequency-range associated with somatosensory feedback to be between 1 and 5 Hz (Fitzpatrick et al., 1992, 1996). Analyses in patients with vestibular, spastic, and cerebellar syndromes led to associate the range <0.5 Hz to visual and vestibular systems (Dupui et al., 1990). The existence of an in-between segment, with no clear-cut defined boundaries—between 0.5 and 1 or 2 Hz—is typically abnormal in case of cerebellar dysfunction. Hence, frequency-based analyses, especially within the range of high-frequencies (proprioception associated with short neural loops) and low frequencies (visuo-vestibular control associated with long neural loops) have opened the door to the quantitative assessment of respective contributions of specific neural loops to postural regulation. Even though authors advise for slightly different ranges over which to apply such analysis, the interest of frequency analysis of postural control is now largely accepted. Comparing eyes opened and eyes closed (Golomer et al., 1994; Davis et al., 2009) in the low frequencies isolates the contribution from visuo-vestibular input, while high frequency analysis of CoP signals in expert vs. novice athletes gauged their reliance on proprioception (Golomer and Dupui, 2000; Paillard et al., 2002). Yet, the interpretation of spectral power in some frequency bands is not straightforward. Although it has been the basis of most studies relying on frequency analysis, it has been suggested that higher power does not necessarily mean lower stability (Dault and Frank, 2004), which could explain inconsistent findings in studies relying on these indicators (Gregoric et al., 1981; van Emmerik and van Wegen, 2002). One first explanation is that CoP movements could be exploratory (Gibson, 1979; Riccio and Stoffregen, 1988), in which case variability would be essential to get information from the environment. Thus, drawing conclusions from one component of the power spectrum is a potential source of error. A more robust approach is to think in terms of strategies of postural control. All in all, the confusion comes from the fact that variability has often been improperly confused with instability (Chagdes et al., 2009; Stergiou and Decker, 2011); both notions are linked but discernable. A second concern may rely on methodology. Algorithms based on Fourier transforms are commonly used for spectral analyses of CoP signals even though CoP demonstrates non-stationary features that prohibit use of such techniques. Power density distribution may then be skewed by unreliable Fourier-based analyses.

There is increasing evidence that physiological systems aiming at maintaining a steady situation, e.g., the postural control system, generate complex fluctuations (Wayne et al., 2013). Consequently, in order to tackle the limitations associated with linear analysis of CoP, non-linear metrics have recently been used successfully. Advantageously, they consider the temporal organization of sway, and are more direct indicators of stability. They bring a holistic view to the analysis of a dynamic control system, and take into account its necessary variations. Among metrics of complexity, the Lyapunov exponent has been used as an indicator of standing stability and a marker of chaos (Collins and De Luca, 1994; Roerdink et al., 2006; Donker et al., 2007; Ladislao and Fioretti, 2007; Lamoth et al., 2009; Lamoth and van Heuvelen, 2012), sample entropy as a measure of randomness (Lamoth et al., 2009; Lamoth and van Heuvelen, 2012), multi-scale entropy has associated sway complexity to stability (Costa et al., 2007; Kang et al., 2009; Manor et al., 2010; Gruber et al., 2011; Wei et al., 2012; Wayne et al., 2014; Zhou et al., 2017), and fractal analyses, mainly detrended fluctuation analysis (DFA), have proven high reliability in measuring scaling in the CoP signal (Collins and De Luca, 1993; Roerdink et al., 2006; Lamoth et al., 2009; Delignières et al., 2011; Kuznetsov et al., 2013; Zhou et al., 2013).

Detrended Fluctuation Analysis (DFA) is a fractal analysis which identifies scaling phenomena in a signal by associating to a scale *n* a fluctuation *F(n)* to finally identify the linear relation between log(*n*) and log(*F*(*n*)) (see Methods). The method can identify short and long-term correlations, as well as anticorrelations. Briefly, it quantifies the *persistence* of a signal. DFA is also particularly interesting since it can identify a certain category of signals called 1/f noise, which is omnipresent in complex systems and human physiology (Diniz et al., 2011; Wayne et al., 2013; Wijnants, 2014). 1/f noise literally means that power density is inversely proportional to frequency. The omnipresence of 1/f noise led to several debates upon its origin in physiological control system. Some authors view this phenomenon as a proper characteristic of physiology and look for a unified origin, idea which can be summarized by the words “1/f scaling is too pervasive to be idiosyncratic” (Kello et al., 2007). Other authors look for specific origins in each physiological subsystem. Despite the ongoing debate on the origin of 1/f noise, authors of both school of thought seem to agree that 1/f noise is the sign of a system which is both robust and adaptable since it is a compromise between rigidity and flexibility. The presence of 1/f noise in the output signal of a physiological system is closely linked to an organized and coordinated control (Marmelat et al., 2012). Thus, unsurprisingly, the presence of 1/f noise has been detected in healthy physiological systems (Goldberger et al., 2002; Hausdorff, 2007; Wayne et al., 2013).

It has been noted that physiological systems can exhibit one, but sometimes even several scalings. Signal integration (summation) or differentiation may be necessary to be able to associate this scaling to 1/f noise (see Method and **Figure 2**), and will be done later in this article for two reasons: first because as explained earlier 1/f noise has been associated with a complex system with interesting properties, and second because the signal analysis using DFA reaches better reliability in such signals than in more persistent ones (Delignieres et al., 2006).

By seeing postural control as the automatic control of the center of mass, the CoP signal displays the control variable, since it is proportional to the ankle torque (Baratto et al., 2002). Analysis of the CoP signal persistence then gives useful information on the control strategy operated to maintain upright posture. Fractal analyses have been applied to CoP signals in the past, leading to different conclusions. Collins et al. (Collins and De Luca, 1993) focused their attention on the position signal and depicted two concurrent phenomena: persistence in the short timescales (<~1 s) and antipersistence in longer timescales (>~1 s), which they interpreted respectively as an open-loop and a closed-loop control (Priplata et al., 2002). It is worth noting that they inferred position-based control of posture, with the closed-loop component triggering at larger timescales because of a threshold. They deduced that the open-loop did not rely on sensory feedbacks, while the closed-loop did (Collins and De Luca, 1994; Priplata et al., 2002). The idea was recently exploited to assess frailty, a situation where both loops were altered (Toosizadeh et al., 2015). Others noted that 1/f noise was observable in long timescales too (Zatsiorsky and Duarte, 2000; Duarte and Zatsiorsky, 2001), and decomposed postural sway in a short scale *trembling* (movements around a reference point), and a larger scale *rambling* (random walk-like movement of said reference point). In 2011, Delignières et al. (2011) pointed a methodological mistake in Collins' work (Collins and De Luca, 1993) indicating that they were actually working on CoP velocity, not on CoP position. Delignières and coworkers differentiated postural trajectories and showed that CoP velocity behaved on the short scale like 1/f noise. This led them to another interpretation: postural control would actually be velocity-based on the short timescales, and the observed crossover between short and long timescales (giving the 1/f noise in the large scales of position) would be due to a boundary effect on velocity leading to strong antipersistence in velocity in the large timescales.

It follows from the above that DFA has proven to be useful for assessing the dynamics of postural control and infer new control theories. Yet, it has until now only been used marginally to distinguish control strategies at specific timescales. DFA has mostly been limited to differentiate two timescale components (Collins and De Luca, 1993; Zatsiorsky and Duarte, 2000; Duarte and Zatsiorsky, 2001; Delignières et al., 2011) with little information about the crossover, and corresponding range of scales. Frequency-based analyses, on the other hand, made the link between CoP signal features and neural loops contribution. Thus, there seems to lack a bridge between frequency analysis and fractal 1/f noise analysis. The aim of the present study was to make a first step in this direction, using a methodology we call Frequency-specific Fractal Analysis (FsFA).

The innovative method (FsFA) we propose here is based on DFA. Therefore, as a first step, we briefly describe DFA developed by Peng and collaborators (Peng et al., 1993, 1994).

## Detrended fluctuation analysis (DFA) and its pitfalls

Detrended Fluctuation Analysis (DFA) was first introduced as an indicator of the presence of a scaling phenomenon (Peng et al., 1993, 1995). The main output of the method is the scaling exponent α. It quantifies the scaling in a signal and is related to the Hurst coefficient H (Hurst, 1956). It is now mainly used to detect long-range correlation. DFA has been preferred over other direct computation of the Hurst exponent or frequency analysis because it allows the detection of long-range correlations embedded in seemingly non-stationary time-series, while avoiding spurious detection of apparent long-range correlations that are an artifact of non-stationarity (Peng et al., 1995).

**Figure 1:**
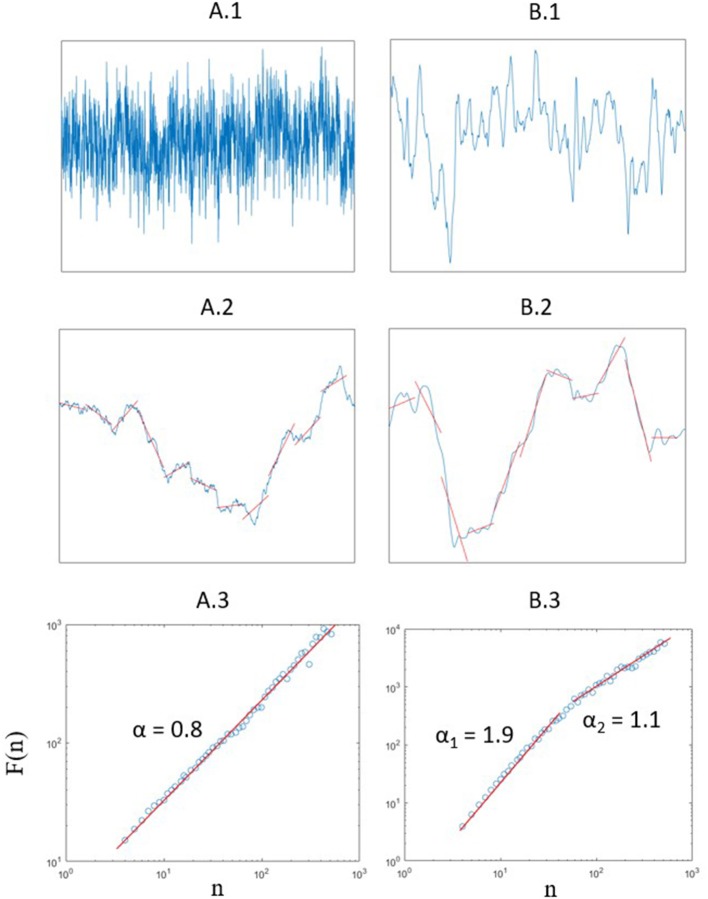
**(A.1)** Artificial fractal signal with α = 0.8 (Generated using Davies and Harte method Davies and Harte, 1987. **(A.2)** Example of a detrending for a fixed value of *n* = 181 of the artificial signal after integration. **(A.3)** Fluctuation function and value of α obtained by the slope of the curve. **(B.1)** Typical position signal obtained for the displacement of COP along the AP axis. **(B.2)** Example of a detrending for a fixed value of *n* = 181 of the typical signal after integration. **(B.3)** Fluctuation function: at least two different scalings are observed here. Note that both DFA show F(n) vs. n in a log-log plot.

DFA algorithm contains several steps (see Figure 1), detailed here for an initial signal x of size N:

1. The signal x is integrated:
  $$\begin{array}{r} y\left(k\right)=\overset{k}{\underset{i=1}{\sum }}\left(x\left(i\right)-\bar{x}\right) \end{array}$$
2. The integrated time-series *y* is then divided into non-overlapping boxes of length n and in each box a straight line is fit to the data using least square approximation (representing the trend in each box). The signal constructed with these lines is called *y*_*n*_.
3. The root-mean-square fluctuation of the integrated and detrended time-series obtained is calculated by:
  $$\begin{array}{r} F\left(n\right)=\sqrt{\frac{1}{N}\overset{N}{\underset{k=1}{\sum }}{\left[y\left(k\right)-y_{n}\left(k\right)\right]}^{2}} \end{array}$$
  - Steps 2 and 3 are repeated for a range of n, usually for *n* = 4 to *n* = N/4.
4. Typically, *F(n)* will increase with *n* as a power law:
  $$\begin{array}{r} F\left(n\right)\sim n^{\alpha }\Rightarrow \log\left(F\left(n\right)\right)\sim \;\alpha \;\times \;log\left(n\right) \end{array}$$

Such a relationship is the marker of a scaling phenomenon. We get the scaling exponent α by the slope of the line of log(*F(n)*) vs. log(*n*) (or the line of *F*(*n*) vs*. n* in a log-log plot, which is strictly equivalent). Note that throughout this article, *log* defines the base 10 logarithm (even though natural logarithm holds for most equations).

Depending on the phenomenon observed, different ranges of box sizes can be selected, which specify a scaling region to be analyzed. It has been remarked that if all the values of *n*∈ ℕ in a certain range are selected, a high concentration of data points will be regrouped in the large box sizes (due to the log plot), giving this zone excessive weight for the linear fit. To cope with that problem, we selected box sizes on a pseudo exponential scale, in a way similar to evenly-spaced DFA (Almurad and Delignières, 2016), meaning that the box sizes *n* were selected to be approximately evenly spaced on the logarithmic plot.

**Figure 2:**
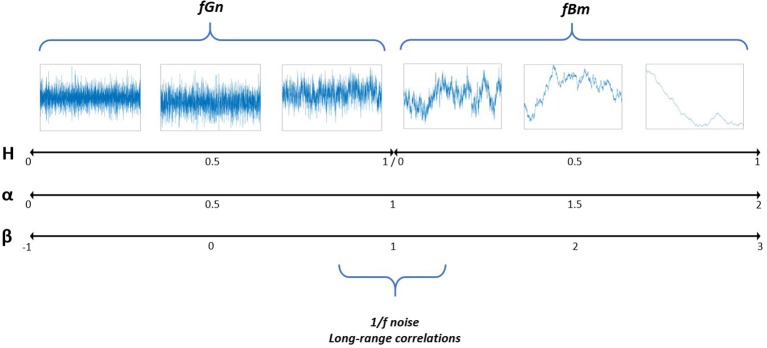
Illustration of the fGn/fBm continuum, with corresponding Hurst exponent H, scaling exponent α and β index. This illustration was largely inspired by Marmelat (Marmelat et al., 2012).

Interpretation of DFA takes root in the fractional Gaussian Noise (fGn)/fractional Brownian Motion (fBm) framework (Mandelbrot and van Ness, 1968), which itself is an extension of Gaussian Noise (uncorrelated white noise), and Brownian Motion (the cumulated sum of white noise, red noise, also called “random walk”). fGn are stationary processes with constant mean and variance, whereas fBm are non-stationary with stationary increments. Differentiating a fBm creates a fGn, and summing a fGn produces a fBm. The related processes are characterized by the same Hurst coefficient. While gaussian noise is uncorrelated and brown noise is strongly positively correlated: the continuum permitted by fGn and fBm covers all kind of correlations (see Figure 2 for an illustration). In the continuum, the definition of H holds an ambiguity at the frontier of fGn and fBm, which would be described as both H = 0 and 1: this is a particular type of signals called pink noise or 1/f noise, which appears in many physiological signals. In the frequency domain, the continuum is characterized by a scaling law where power is proportional to f^−^. This law is revealed by the log-log representation of the power spectrum fitted to a linear relation of slope –ß. When ß equals 1, the signal property is f^−1^ so-called 1/f noise (Figure 2). 1/f noise more largely describes signals with α close to 1, and the term “fractal” is now often used to describe 1/f noise (Mandelbrot, 1975).

The relation between above estimators, scaling exponent α, Hurst exponent H, and “spectral” slope ß, in the limit of infinite time-series is as follows:

(4) $$\{\begin{array}{c} for\;a\;fGn:\;\alpha =H=\;\frac{\beta +1}{2}\\ for\;a\;fBm:\;\alpha =1+H=\;\frac{\beta +1}{2} \end{array}$$

DFA has been shown to be theoretically equivalent to β when analyzing signals of infinite length without noise. It has been shown to give similar results in long signals (Heneghan and McDarby, 2000), but DFA has been preferred for shorter signals closer to 1/f noise (Delignieres et al., 2006). Therefore, we focused on DFA here. However, we should keep in mind the strong link between DFA and a simple frequency analysis, especially in the context of the present paper, where we attempt to make a bridge between frequency and scaling approaches of CoP signals to gain an improved understanding of postural control strategies.

As explained above, the main output of DFA is the scaling exponent α. However, the coefficient itself is often insufficient to draw definitive conclusions, because a unique slope of log(*F*(*n*)) vs. log(*n*) can always be fitted with fairly good approximation, no matter the real shape of the curve. It has often led authors to jump to the conclusion that a signal is 1/f noise, when the truth was actually more complicated. Efforts have been made to warn about this pitfall and to provide tools to avoid flawed conclusions. As an introductive example, the “relative roughness” introduced by Marmelat et al. (2012), takes as exclusion criterion a ratio of local on global variation, which can exclude signals that are too smooth to be considered as fractal. However, unlike mathematical models, 1/f noise in real life signals is necessarily bounded upon a frequency range, delimited at least by physical limits. We think that a careful examination of the log(*F(n)*) vs. log(*n*) plot of DFA, enhanced with considerations on the nature of the signal of interest, can lead to an even better interpretation. Indeed, as α is a scaling exponent, the frequency ranges over which a certain type of scaling is observed is of utmost importance (Figure 1). For example, the determination of the scaling exponent α by the DFA method depends on the sampling frequency of the signal. Would the sampling frequency be an order of magnitude higher than the highest frequency in the analyzed phenomenon (say a 2 kHz sampling of the CoP velocity), DFA would give unreliable results, only dependent on the measure instrument in the smallest boxes. Relative roughness would simply exclude the signal as a potential fractal, fulfilling its role as an exclusion criterion, even though it could contain valuable information if analyzed at the correct scales. This means that a bad-conditioned problem can undermine DFA. In our example we would have to calculate α from box sizes large enough to make physiological sense. We recommend this reflection to be made before any DFA, as it would be made for any interpretation of a power spectral density analysis. It is likely the reason why Kuznetsov et al. (2013) notice a particular slope on a narrow range of small boxes in its Adaptative Fractal Analysis (AFA, a derivative of DFA) on their analysis of postural control at 100 Hz.

More importantly, several fractal behaviors can occur at different timescales, leading to the appearance of different slopes in DFA (Figure 1), each with its own significance. When the case presents itself, the usual reaction is to remark a crossover point in the plot, then split the analysis *a posteriori* in two ranges on either side of the crossover, and report a short-range and a long-range scaling exponent. Depending on results consistency, the ranges of analysis can be fixed with sufficient empirical background. This has been the case for example in the fractal analysis of heart rate variability, where common practice is to compute two scaling exponents, for short and long-term correlations (Peng et al., 1993; Iyengar et al., 1996). Again, we are convinced that there is a need to explicitly clarify the relation between frequency and DFA box-size to shed light on the underlying physiology.

Here we propose a methodology for CoP analysis allowing us to apply DFA with the concern of associating objectively scaling ranges with frequency ranges, since they seem to have physiological meaning.

## Study 1: development of a frequency-specific fractal analysis

### Material and methods

All computations in this article were done in Matlab (Matlab R2016b, Mathworks), using available functions. The following lines aim at providing the foundations of a method we call FsFA (Frequency-Specific Fractal Analysis) by making plain the relation between a box-size in DFA and a frequency. The full algorithm we purposely developed for the present study, called FsFA, is available on mathworks.com.

The existence of a correspondence between DFA box-size and signal frequency offers two advantages: for signals which fractal component(s) are not *a priori* known, it serves as a tool to better interpret the graph of log(*F*(*n*)) vs. log(*n*) prior to the computation of the scaling exponent α. For signals which fractal components are known, it can help to define one or several α calculated over specific ranges of DFA box-sizes.

Some authors have implicitly associated box-size and signal frequency components in the past when analyzing human standing (Duarte and Zatsiorsky, 2001) or heart rate variability (Schmitt and Ivanov, 2007). They assume that some typical frequency components (f) are represented in boxes of given size (*n*) in the DFA analysis based on the implicit assumption that $n\;=\;\frac{1}{\left(\frac{f}{f_{s}}\right)}$ where *f*_*s*_ is the sampling frequency. Although this can seem logical, to the best we know there exists neither theoretical nor empirical background to support this relationship.

**Figure 3:**
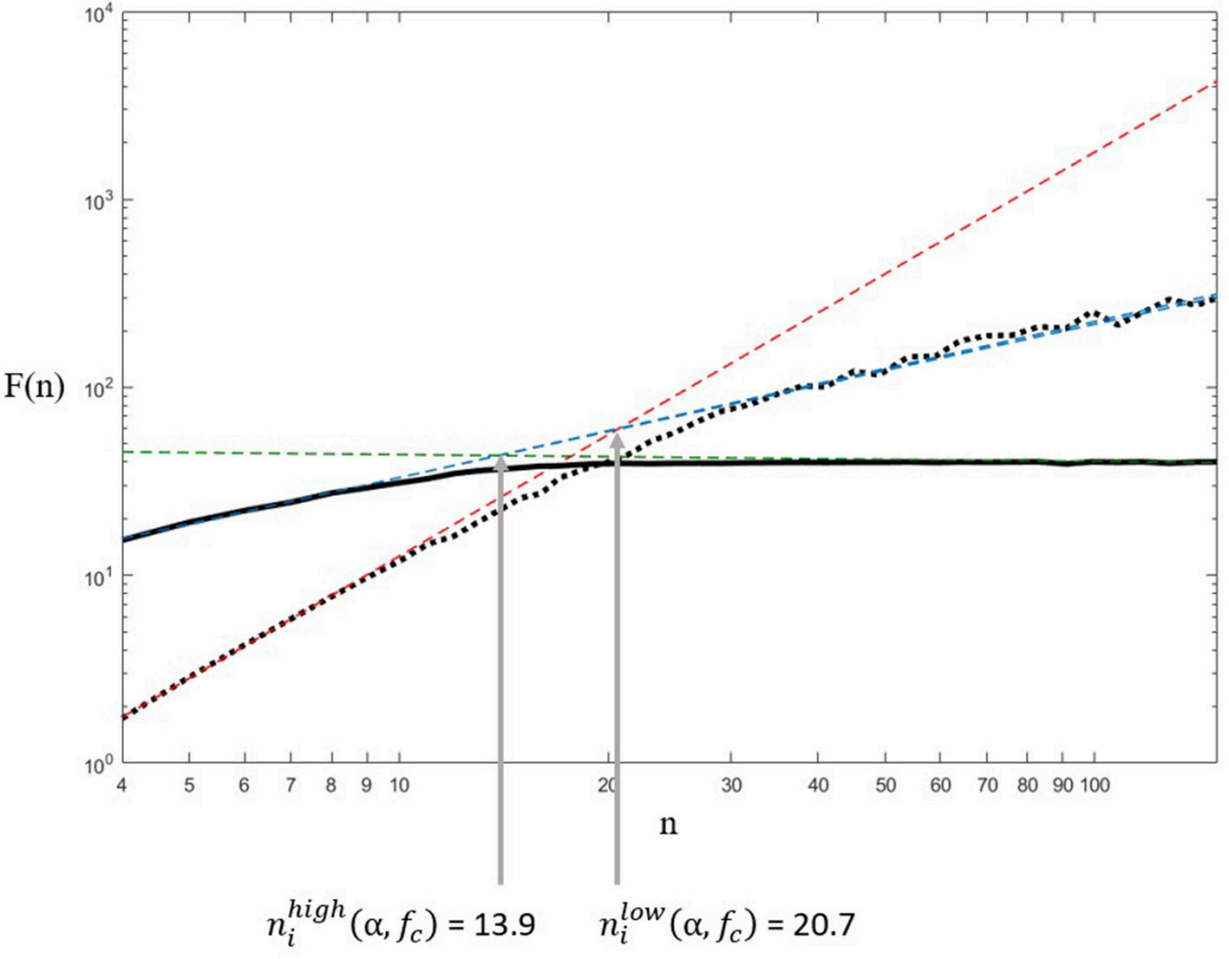
Example of the estimation of the cutoff box of $n_{i}^{low}\left(\alpha ,\;f_{c}\right)$ and $n_{i}^{high}\left(\alpha ,\;f_{c}\right)$, done in the low-passed signal $X_{i}^{low}\left(\alpha ,\;f_{c}\right)$ (thick black dotted line), and high passed signal $X_{i}^{high}\left(\alpha ,\;f_{c}\right)$ (thick black full line). In this example, we chose $X_{i=1}^{low}\left(\alpha =0.8,\;f_{c}=0.056\right)$. The crossover was considered to be at the intersection of the asymptotes in each signal (thin dashed straight lines). Note that the asymptote of $X_{i}^{low}\left(\alpha ,\;f_{c}\right)$ for high values of n and the asymptote of $X_{i}^{high}\left(\alpha ,\;f_{c}\right)$ for low values of *n* (thin dashed blue straight lines) are perfectly superimposed since they correspond to the unaltered frequency ranges of the original signal X_i_(α) (not plotted here for clarity), which has a unique scaling, hence a unique slope.

Here we purposely developed a specific simulation-based analysis in an attempt to clarify the relationship between DFA box-size and frequency component in several steps:

1. We generated artificial fGn signals using Davies and Harte method (Davies and Harte, 1987; Bardet and Bertrand, 2007), of length 2048, ranging for α = 0.1 to α = 0.9 with steps of 0.1. Ten signals were generated for each value of α. Let us call these signals X_i_(α) for i = 1 to 10.
2. These signals were then filtered, first with a bank of zero-lag first-order *low-pass* Butterworth filters of cutoff frequencies f_c_. Symmetrically, they were filtered with a bank of zero-lag first-order *high-pass* Butterworth filters of cutoff frequencies f_c_. Let us call the obtained signals $X_{i}^{low}\left(\alpha ,\;f_{c}\right)$ and $X_{i}^{high}\left(\alpha ,\;f_{c}\right)$. Here sampling frequency is considered f_s_ = 1, which is equivalent to saying that the studied frequencies correspond to frequencies normalized by the sampling frequency f_s_. The values of f_c_ were chosen so that $\frac{1}{f_{c}}$ ranged from 4 to 200 with steps of 1: we chose this range because if $n=\;\frac{1}{\left(\frac{f}{f_{s}}\right)}$, in agreement with other authors, the corresponding box sizes in DFA would range from the classically admitted minimum 4 to ~N/10 which allows us to avoid spurious effects in boxes close to N/4. Filtering of signals with α>1 with Butterworth filters is not allowed since such signals are non-stationary, which is why we restrained our analysis to signals with α < 1 (fractional Gaussian noises).
3. Such a filtering generated a cutoff in the DFA plot of log(*n*) *vs*. log(F(n)) (Figure 3). We automatically identified the box sizes where the cutoff happened in filtered signal for each f_c_, $n_{i}^{low}\left(\alpha ,\;f_{c}\right)$ and $n_{i}^{high}\left(\alpha ,\;f_{c}\right)$, respectively for the low-passed and high-passed signal. The cutoffs were identified as the intersection of the high frequency and low frequency asymptotes, using the following assumptions:
  - Unaffected frequencies (low frequencies for a low-pass filter, and high-frequency for a high-pass filter) present a scaling identical to the original signal, so the asymptote on this part has a slope of α.
  - High-frequencies filtered by a low-pass signal present a slope of 0.
  - Low-frequencies filtered by a high-pass filter present a slope of α+1. Indeed, first-order filtering of a fractal signal increases the value of β by 2, so $\alpha_{filt}=\frac{\beta_{filt}+1}{2}=\frac{\beta +3}{2}$ = α+1.
  - The “cutoff boxes” were then defined as the mean of the high pass cutoff box size and low pass cutoff box size:
    $$\begin{array}{r} n_{i}^{\alpha }\left(\;f_{c}\right)=\;\frac{n_{i}^{low}\left(\alpha ,\;f_{c}\right)+\;n_{i}^{high}\left(\alpha ,\;f_{c}\right)}{2} \end{array}$$
  - These cutoff boxes were considered to be the box sizes corresponding to the cutoff frequency. This gives a relationship between f_c_ and the cutoff box for each α, obtained for our 10 simulated signals X_i = 1..10_(α) for each α value (step 1).
4. We hypothesized that $n_{i}^{\alpha }\left(\;f_{c}\right)$ was a linear function of $\frac{1}{f_{c}}$ with no offset, since $n_{i}^{\alpha }\left(\;f_{c}\right)$ should be proportional to ${\frac{1}{f_{c}}}_{.}$ We then identified by least square approximation $a_{i}^{\alpha }$ such as:
  $$\begin{array}{r} n_{i}^{\alpha }\left(\;f_{c}\right)=\frac{1}{f_{c}}\times a_{i}^{\alpha }. \end{array}$$

### Results

**Figure 4:**
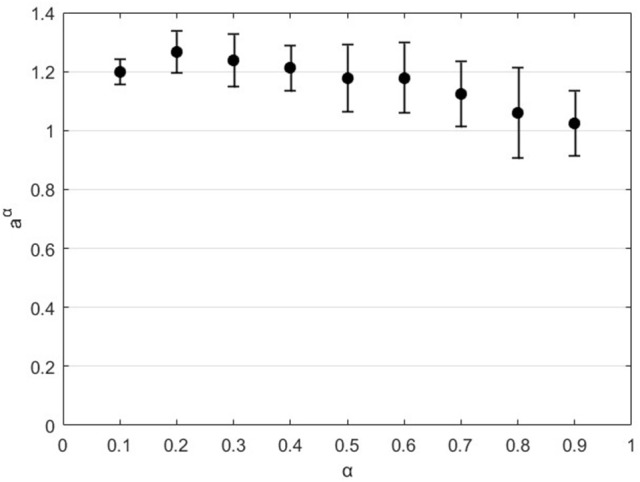
Estimation of the slope *a*^α^. Values are mean ± SD from *n* = 10 simulated signals for each value of α.

A typical outcome of the simulation procedure is illustrated in Figure 3. It appears clearly that the filtering of synthetic fGn signals generated a crossover in the log(*F*(*n*)) vs. log(*n*) relationship established with DFA. Each crossover was assessed by computing the intersection of both asymptotes of each signal. In the example of Figure 3, the high pass filter generated a crossover at *n* = 13.9 while the low pass filter on the same signal generated a crossover at *n* = 20.7. The average value between *n* = 13.9 and *n* = 20.7, i.e., *n* = 17.0 was considered to be the box size corresponding to the cutoff frequency used in low pass and high pass filtering (f_c_ = 0.056 Hz). The repetition of this procedure for f_c_ = 0.005 to 0.25 Hz combined with α = 0.1 to 0.9 and 10 simulated signals per α value, provided 10 linear approximations for each α, which slopes are reported in Figure 4. The linear approximations were computed for 8 < T_c_ < 43 (0.0233 < f_c_ < 0.125) since this range presented proper affine shape. Variations outside this zone may be due to bad identification of the cutoff by our algorithm, or by finite size and sampling frequency limits of the filters we used. We will then assume that the relation holds for any *n* and *f*_*s*_ despite the approximation being made locally. Considering reasonably weak variations both within and between slope values as illustrated in Figure 4, we conclude that the direct relation that was assumed in the literature was correct:

$$\begin{array}{r} n\left(f_{c}\right)=\frac{f_{s}}{f_{c}} \end{array}$$

Therefore, we have now all the necessary elements to analyze scaling phenomena on frequency ranges of interest, by computing DFA on box sizes comprised in said ranges: we call this general method Frequency-Specific Fractal Analysis (FsFA). When applied to CoP signal analysis, it follows that we can estimate box size ranges within which scaling exponents can be computed, in association with typical frequency bands wherein proprioceptive and visuo-vestibular loops are thought to operate (Table 1).

**Table 1:** Frequency ranges for using DFA on specific control loops of posture.

	**Proprioception-associated range**	**Visuo-vestibular associated range**
Frequency	20–2 Hz	0.5–0 Hz.
Associated box size	2–20	80–∞
Limits for DFA	4–20	80–N/4

*As DFA is generally limited to box sizes from 4 to a quarter of the total length of the signal (here 512), the last row displays the real chosen range of boxes*.

It is worth noting that box sizes definition in FsFA, while bounded by the frequency-range of interest, were selected to be a geometric sequence, so that their log is an arithmetic sequence. This choice was made to follow recommendations of evenly-spaced DFA (Almurad and Delignières, 2016). The sequence was defined as follows:

- *n*(1) is defined by the minimal frequency chosen
- *n*(2) = *n*(1)+1
- $for\;i>2,\;n\left(i\right)=n\left(i-1\right)\times \frac{n\left(2\right)}{n\left(1\right)}$

## Study 2: FsFA application to disturbed posture

### Material and methods

Prior determination of the relationship between signal frequency components and DFA box size based on synthetic signals made it possible next to apply the freshly elaborated method FsFA to CoP recordings during standing in disturbed conditions. For that, eight female rugby players (age 20.3 ± 1.1 years, mass 63.2 ± 6.5 kg, height 164.3 ± 6.4 cm) gave their informed consent to participate in the study. They were asked to maintain successively quiet stance on a platform with: (i) eyes opened (reference); (ii) eyes closed (visual inputs disturbed); and (iii) eyes opened after intense exercise (somatosensory inputs disturbed). This experiment was designed to analyze the impact of altering individually two sensory feedback loops. The study supports the principles of the Declaration of Helsinki and was approved by the institutional review board of the faculty. The platform used (Winposturo, Medicapteurs, Balma, France) is equipped with three strain gauges to compute the position of the center-of-pressure. The CoP signal was recorded at sampling frequency 40 Hz for 51.2 s, which gave a total of 2.048 data points. While standing, participants held their arms alongside their body and focused on a cross on a wall 3 m ahead. Their feet were oriented 15° from the sagittal midline with heels 4 cm apart. Prior familiarization to the test was allowed. Fatigue arose after an intermittent all-out exercise made of jumps, hopping, stepping and short sprints. The main expected effect is a reduced contribution of muscle spindles to the proprioceptive input flow (Brockett et al., 1997; Thedon et al., 2011).

We propose that both frequency and scaling analyses that have been conducted in the literature focus on the same frequency-range and are two complementary analyses of the same neural systems operating in postural control. Indeed, Collins and collaborators positioned the crossover in DFA of posture around f = 1 Hz (Collins and De Luca, 1993). Delignières and collaborators had similar results, since we see in one of their graphs a crossover around log(n) = 1.8 for data sampled at 40 Hz (Delignières et al., 2011), which we will see using Equation (6) can be translated to f = 0.63 Hz. This way, we chose to assess both spectral power and frequency-specific scaling exponents, on the range of frequency 0 to 0.5 Hz to examine visuovestibular-based control, and 2 to 20 Hz to examine proprioception-based control. We have mentioned that no consensus has yet been found as exactly what frequency-ranges are “best” to analyze: we chose those because they let an equal space (on a log plot) between Collin's 1 Hz crossover and the first frequencies analyzed around (0.5 and 2 Hz), which can account for a transient zone between both scalings.

We analyzed fluctuations of both CoP position and CoP velocity (derived from position) on AP axis. Power spectral density was computed using Discrete Fourier Transform. Scaling analysis was performed with DFA as described above. Thanks to FsFA, we got two scaling exponents α from two specific ranges of box size that corresponded to selected signal frequencies (Table 1).

### Results

**Figure 5:**
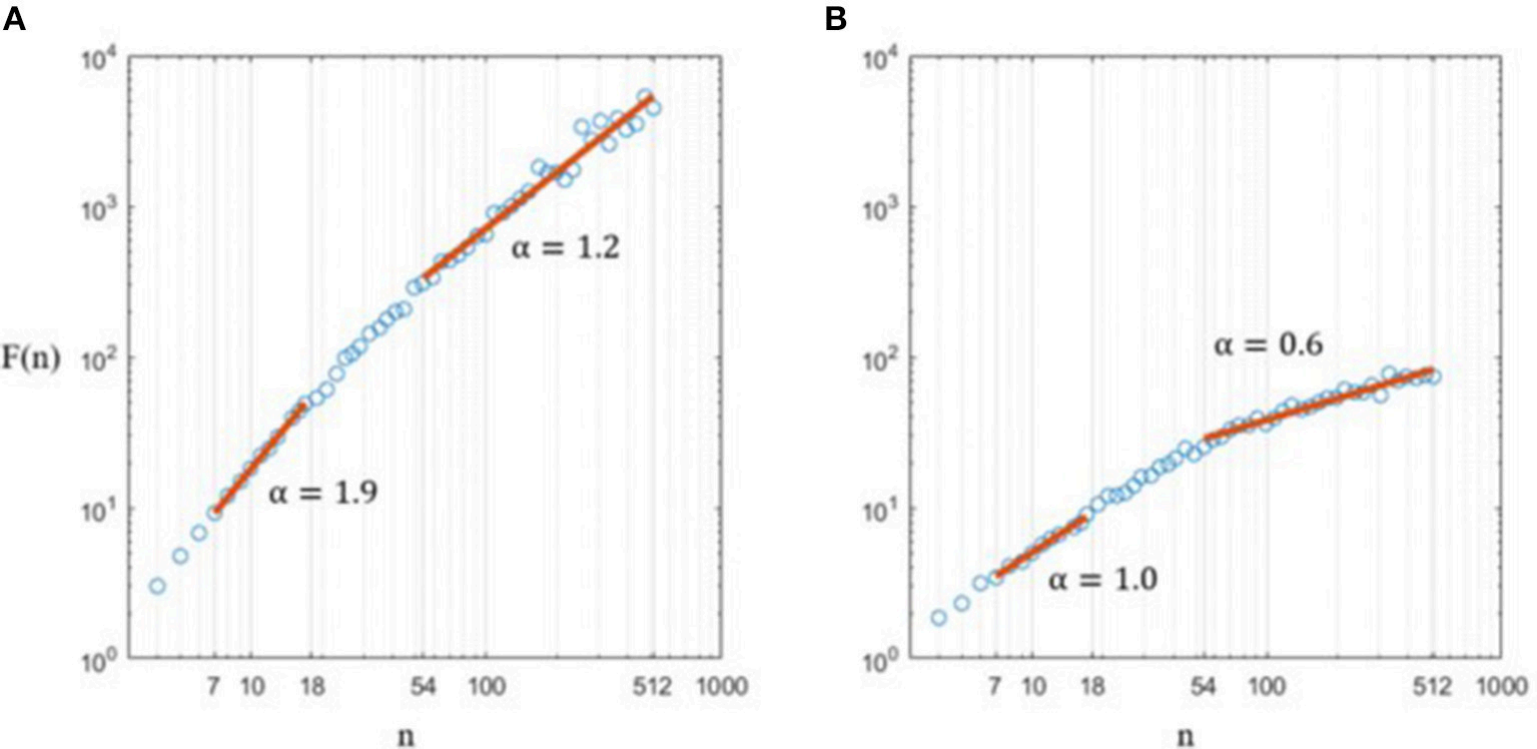
Calculations of scaling exponents α on a typical CoP displacement along the AP axis **(A)** and of the same signal, derived **(B)**.

First, the absence of a unique slope in log(*F(n)*) vs. log(*n*)—classically reported in the literature—was visually confirmed here in both CoP position and CoP velocity signals (Figure 5). We computed scaling analyses within two ranges guided by frequency boundaries (Table 1): (i) short timescales, defined by 4 < *n* < 20 and associated with high frequencies (2–20 Hz), the range wherein proprioceptive control has been shown to dominantly operate; and (ii) long timescales, defined as *n* > 80 and associated with low frequencies (0–0.5 Hz) wherein visuo-vestibular control has been shown to dominantly operate.

#### Short timescales—high frequencies

In the reference state (eyes open, unfatigued), over short timescales, individual scaling exponents points to the presence of a scaling close to 1/f noise in CoP velocity (α = 1.30 ± 0.16) but not in CoP position (α = 1.93 ± 0.04). Therefore, only α for CoP velocity was considered for the subsequent analysis in short timescales (i.e., high frequencies), where postural control at rest is compared to control after fatiguing exercise.

**Figure 6:**
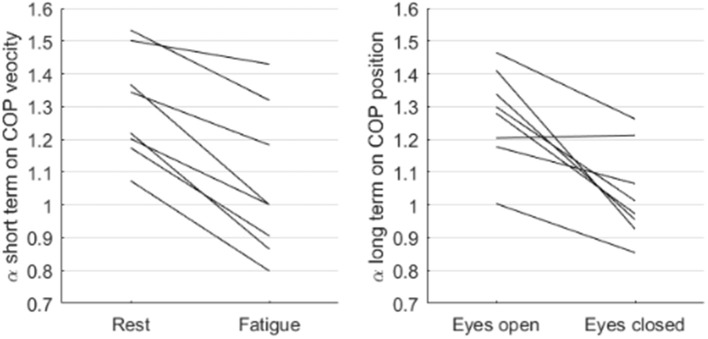
Individual changes in short term scaling exponent on CoP velocity and long-term scaling exponent on CoP position, respectively with fatigue and eye closing, for all subjects.

Intense exercise led to an increase in spectral power of CoP in the high frequencies (0.051 ± 0.037 to 0.117 ± 0.040 mm^2^, *P* < 0.01). A significant decrease in the scaling exponent α was observed (1.30 ± 0.16 to 1.06 ± 0.23, *P* < 0.001). Scaling exponent of all subjects are reported in Figure 6.

In the reference state, there was no significant correlation between spectral power and scaling exponent related to scales associated with proprioception. This absence of correlation between individual spectral power and scaling exponents was confirmed in the fatigued state. This result suggests that these two metrics are likely sensitive to distinct control properties.

#### Long timescales—low frequencies

In the reference state, over long timescales, individual scaling exponents point to the presence of a scaling close to 1/f noise in CoP position (α = 1.27 ± 0.15) but not in CoP velocity (α = 0.39 ± 0.10). This is the opposite scenario of what is described above for short timescales. Taking fractal properties in CoP position and not CoP velocity, only α for CoP position was considered for the subsequent analysis in long timescales, where eyes closed was compared to eyes open during quiet standing.

The disturbance of visual inputs due to the eyes closing did not modify spectral power of CoP position significantly in the associated frequency band (14.4 ± 10.2 vs. 9.3 ± 5.5, *ns*) over these scales, but changed the scaling exponent (α = 1.27 ± 0.15 vs. 1.03 ± 0.14, *P* < 0.01). Scaling exponent of all subjects are reported in Figure 6.

In the reference state, there was a poor but significant correlation between spectral power and scaling exponents in this frequency range (*p* = 0.037). No significant correlation was found between spectral power and scaling exponent in the eyes closed situation. This result suggests that these two metrics are likely mostly sensitive to distinct control properties.

## Discussion

In the present study, we hypothesized that scaling analysis of CoP dynamics may benefit from taking into account physiological backgrounds classically associated with the frequency components of the CoP signal, to uncover postural control strategies. To this end, we developed Frequency-specific Fractal Analysis (FsFA).

First, based on simulated signals, we established for the first time a relationship between DFA box size and frequency components in a signal; by doing so, we laid the foundations of FsFA. We then applied the method to the analysis of the CoP in order to demonstrate how it could be useful in the systematic assessment of frequency-specific scalings.

### Coherence with previous works

In agreement with previous studies, FsFA of CoP fluctuations during quiet standing showed a crossover phenomenon and two scaling regions (Figure 5), which is definitely a hallmark of postural control (Collins and De Luca, 1994; Zatsiorsky and Duarte, 2000; Duarte and Zatsiorsky, 2001; Priplata et al., 2002; Delignières et al., 2011). Classically, both scaling exponent are computed based on pure mathematical considerations, without physiological background: only visual inspection of the DFA log(*F(n)*) vs. log(*n*) plot guides the computation of scaling exponents. To the best we know, neither clear boundaries nor recommended localization of the crossover are defined in the literature. Here, we suggest that using what we called *Frequency-specific Fractal Analysis* (FsFA), the two scaling exponents might be better assessed by computing two distinct linear fits of log(*F*(*n*)) vs. log(*n*), guided by frequency bands of interest. Based on the bandwidth 2–20 Hz wherein spectral power is dominantly influenced by proprioceptive short neural loops, the short-range scaling exponent was computed within the DFA box size range 4 < *n* < 20 (Equation 7). On the other hand, the long-range scaling exponent was assessed over the range 80 < *n* < N/4, guided by frequencies < 0.5 Hz classically associated with visuo-vestibular neural loops (see Table 1).

The frequential view, which brought us to analyze scaling below 0.5 Hz on one side, and above 2 Hz on the other side, is coherent with the scalings previously observed by authors. Indeed, Collins and collaborators positioned the crossover in DFA of posture around f = 1 Hz (Collins and De Luca, 1993), and Delignières et al. pointed to a crossover around log(n) = 1.8 for data sampled at 40 Hz (Delignières et al., 2011), which can be translated to f = 0.63 Hz thanks to present simulations (Equation 7). The added value of the present study clearly does not lie in the existence of two scalings in postural control, which has already been observed before, but in the evidence that both scalings vary as a function of sensory input disturbances (Figure 6).

### Scaling phenomena in postural control

Guided by the localized presence of 1/f noise-like behavior, CoP velocity was studied in short timescales and CoP position in long timescales. As mentioned in Introduction, this choice was driven by two arguments: first, 1/f noise has been associated with a complex system with interesting properties, and second because DFA reaches better reliability in such signals. As we based our analysis on classically admitted frequency bands of postural control, we expected short-term CoP scaling properties to be linked to proprioceptive inputs and long-term CoP scaling properties to be linked to visuo-vestibular sensing.

Assuming that scaling exponents determined by FsFA are pertinent indicators of postural control strategies, then those indicators should be sensitive to sensory disturbance. By alternatively fatiguing leg muscles and suppressing visual inputs, subtle changes in postural strategy were unraveled here in athletes by FsFA. Velocity scaling (short timescales) as well as position scaling (long timescales) shifted from a persistent signal (α~1.3) to a less persistent signal (α~1) after specific disturbance (respectively, fatigue and eyes closed). In our conditions, these shifts in scaling demonstrated poor (if any) reliance on shifts in frequency markers, pointing to distinct physiological backgrounds. This way, for both velocity and position CoP signals, the drop in scaling exponent observed here seems to be a strategy independent from the classically observed increase in power in each frequency band.

In the present study, when perturbing each input, it seems that the strategy chosen was to adopt a tighter control, with less tolerance to big variations, thus favoring higher-frequency control, closer to the equilibrium, rather than low-frequency control allowing for slow variations outside equilibrium. A similar strategy was observed by Dingwell and Cusumano (2010) for stride speed fluctuations when walking on a treadmill: a diminution of persistence (i.e., drop in scaling exponent) was shown when speed was constrained by a treadmill, which they associated with “cautious gait”, an overcompensating strategy. As well, Terrier and Dériaz (2012) inferred a tighter control from a drop in scaling exponent of inter-stride intervals, when an auditory cueing served as constraint. Though the change they revealed were more radical since the observed variables (inter-stride interval or gait speed) changed from a persistent signal (α > 0.5) to an antipersistent one (α < 0.5), a similar interpretation can be made here with a “cautious postural control”, with a subtler change in scaling exponent.

As both changes in strategy operate within a typical frequency range defined in postural control with only poor change in total power, the phenomenon, which gives us useful insight for the understanding of postural control strategies, escapes detection by classical Fourier analysis. The valuable information in this scenario arises only from Frequency-specific Fractal Analysis (FsFA) of postural control.

### Origin of scalings

The pervasiveness of fractals in anatomical structures as well as in physiological time series has led number of authors to explore potential sources of such signals. In the case of postural control, we support the idea that those signals are coming from a specific distribution of intrinsic timescales within and between neural loops (Pellet et al., 1970-2013; West and Shlesinger, 1989; Sabatier et al., 2015). Indeed, a fractal signal, by definition, does not have one characteristic timescale and can only be described by a repartition of timescales: this is equivalent to saying that there is a distribution of frequencies, as seen previously with power spectral density. Possessing a great number of timescales is necessary but not sufficient to generate fractal signals (white noise also contains all frequencies). The specific organization of these timescales in postural control may come from an auto-organization of involved neural networks, originating from the numerous internal degrees of freedom and interactions within and between network's components. Such behaviors have been described in many other fields (West and Grigolini, 2011) and often called self-organized criticality (Bak et al., 1987; Jensen, 1998). Similarly, many authors have linked complexity (quantified either by proximity to 1/f noise or entropy) to a property well-known in biology called degeneracy, the capacity of the system to perform the same function with elements that are structurally different (Tononi et al., 1999; Edelman and Gally, 2001; Whitacre and Bender, 2010; Delignières and Marmelat, 2013). Both views share strong similarities, and both make sense from an evolutionary point of view (Whitacre and Bender, 2010). In particular, fractal-like systems have shown several advantages in automatics for dynamical control theory (Sabatier et al., 2015), which can be transferred to postural control. In our case the system seems to have two such organized subsystems as shown above.

These numerous timescales can be operated by the human body because it possesses different intrinsic timescales due to its complex organization (Wayne et al., 2013). In the case of postural control, each subsystem we defined (proprioceptive and visuo-vestibular) relies on various timescales. For example, proprioceptive inputs feed short loops involving spinal motoneurons. In the spine, each afferent neuron has a specific length which depends notably on the distance between the sensor and the synapse, and each efferent motoneuron has a specific length which can depend on the distance between the synapse and the actuator. The place of the sensor, the number of synapses, and the position of the actuator are only a few among the many factors that impact the delay in signal transmission. When multiplied by the number of afferent and efferent structures involved in postural control, a distribution of timescale is created, which may be the source of the fractal signature in the CoP signal.

In short, the two scalings emerge each from a distribution of complex neural control loops, organized in two distinct subsystems, which operate over specific timescales. Local scaling exponents are putative markers of the respective contribution to overall control strategy by the two distinct subsystems.

In each scaling, the change in scaling exponent with sensory perturbation would then be due to a reorganization of the frequency distribution implied in control. We can then hypothesize that when a subsystem is perturbed (via fatigue or deprivation of sensory input in our case), the many internal loops from sensors to actuators which are distributed on various timescales undergo modifications that change their distribution. This redistribution shows both a decreased power in lowest frequencies and increased power in highest frequencies in the concerned frequency band, which would generate the change in power spectral density slope, hence the drop of scaling exponent, as observed here.

### Interpretations and confrontation with other theories

Our results agree with observations made by previous authors and provide additional supports to some conclusions.

Previous works provided theories on the meaning of the two scalings. In the rambling-trembling framework, in light of tangential forces analysis on a force platform, short term scaling was associated with “trembling” and long-term scaling to “rambling” (Zatsiorsky and Duarte, 2000; Duarte and Zatsiorsky, 2001). We hypothesized that short term scaling—trembling—could be associated with short neural loops with proprioceptive inputs, while long term scaling—rambling—could be associated with long neural loops with visuo-vestibular inputs. The changes in scaling exponent due to sensory disturbance, reported here in study 2, support that view.

**Figure 7:**
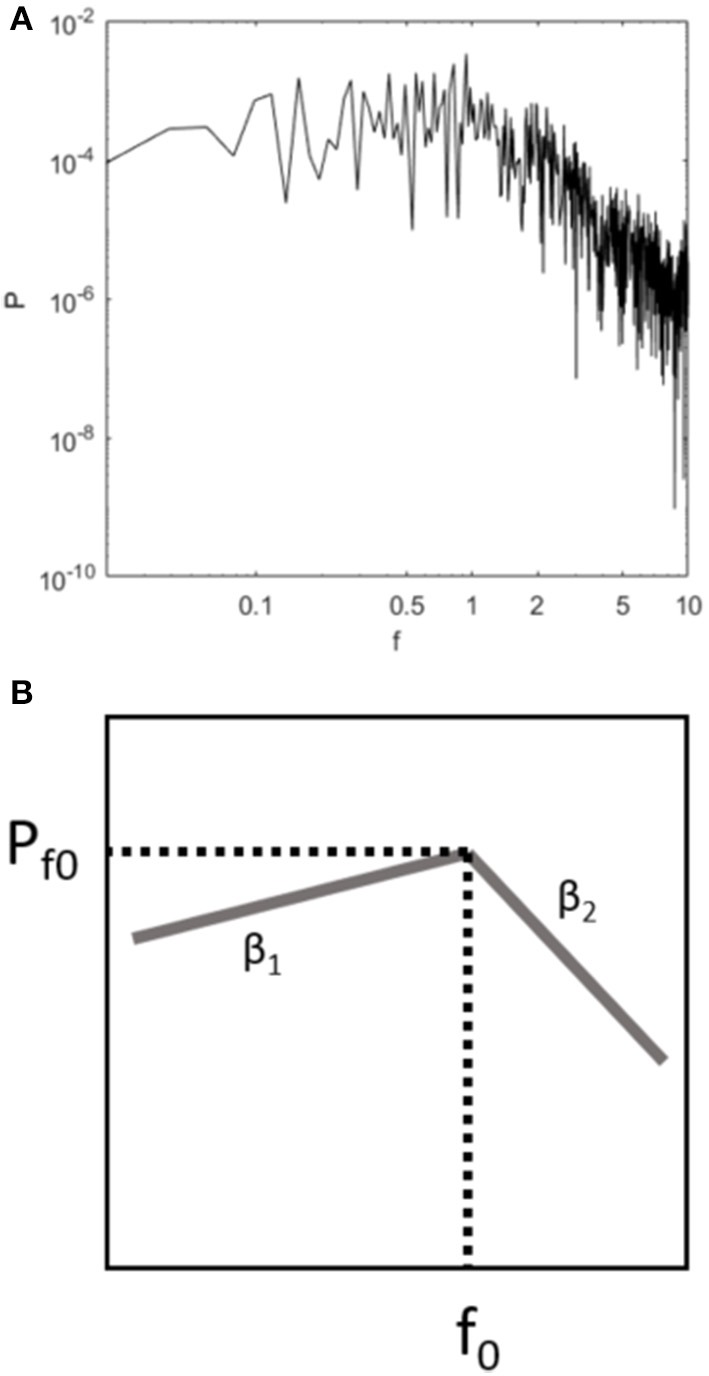
Typical PSD of COP velocity on the AP axis **(A)**, and its schematic representation **(B)** revealing four key parameters: P_f0_, β_1_, β_2_, f_0_.

In the works of Delignières et al. (2011), the scalings' crossover was seen as a consequence of a bounded velocity. Witnessing that velocity seems bounded in CoP signals, they inferred that CoP control is mostly based on correcting impulses when a maximal velocity is reached, and that the average absolute maximal CoP velocity (AAMV) is an interesting marker of postural control. We think the approach in Delignières et al. (2011) and the one in the present work are not necessarily exclusive. Rather, they may provide complementary markers from which an improved understanding of postural control strategy could be gained. Indeed, as CoP velocity exhibits a crossover from short term persistent to long term antipersistence, its PSD naturally possesses a maximal value at this crossover around f_0_ (classically around 1 Hz), as illustrated in Figure 7. This maximum value P_f0_ corresponds to AAMV so that $P_{f0}=\frac{AAMV^{2}}{2}$ (though AAMV may be a more accurate estimator of the real value). From this, if we simplify the PSD content by two linear zones, and consider the frequency-range of 0.5 to 2 Hz to represent only a transient zone in the continuity of the two scalings explored above, we have an even simpler definition of the PSD content. Indeed, Figure 7 shows that the whole frequency content of the CoP velocity signal could then be described by four parameters:

- P_f0_ (or AAMV)
- β_1_, the opposite of the slope of the low-frequency antipersistent range, with
  $$\begin{array}{r} \beta_{1}=2\times \alpha_{1}-3 \end{array}$$
- α_1_ being here the frequency-specific fractal exponent associated with visuo-vestibular loops (low-frequencies) in CoP position. This result is obtained by the fact that β_1_ is computed from the CoP velocity while α_1_ is computed from CoP position, its integral, so more precisely: β_1_ = (2 × α_1_ − 1) − 2.
- β_2_, the opposite of the slope of the high-frequency persistent range, with
  $$\begin{array}{r} \beta_{2}=2\times \alpha_{2}-1 \end{array}$$
- α_2_ being here the frequency-specific fractal exponent associated with proprioceptive loops (high-frequencies) in CoP velocity.
- f_0_, the crossover frequency, which might be variable from one individual to another but is likely between 0.5 and 2 Hz according to the literature.

This way, without presupposing any causality between AAMV and the existence of a crossover between short-term and long-term scalings, we can simultaneously make all parameters appear in one model. It should also be noted that these four parameters contain all the information on the total power in fixed frequencies as classically done in the literature. This new theory would be an interesting framework to analyze CoP signals in future works.

### Limits and perspectives

FsFA could certainly benefit from future improvements. The relation between a specific frequency and the box-size in DFA analysis, which is the cornerstone of FsFA, still lacks a theoretical proof which could give it better reliability. Other researchers may find it interesting to explore mathematical proof of the equation.

Potential extensions of FsFA can be imagined using other fractal analysis methods. For example, Adaptative Fractal Analysis (AFA) is a fractal analysis method very similar to DFA which, if it still not has been used as often as DFA, presents several advantages over it (Gao et al., 2012; Riley et al., 2012; Kuznetsov et al., 2013). AFA could benefit from a similar frequency interpretation. We can also imagine a frequency-specific multifractal analysis, which could for instance be a direct extension of FsFA by using multi-fractal DFA (Kantelhardt et al., 2002; Matic et al., 2015), or an indirect extension using multifractal crossovers (Nagy et al., 2017). Future research may focus on developing and testing the efficiently of such tools.

By choosing to follow frequency bands accepted for spectral analysis, we are using a framework which has been constructed essentially for spectral analysis. To build a complete theory of postural control, with variable fractional differentiator-based control loops, identified via frequency-specific scaling, future research may try to better define proper frequency bands adapted to this analysis. To this end, a potential lead would be to individually degrade specific parts of a subsystem, fatiguing thigh muscles for instance, and control how the scalings locally vary. This kind of study would allow for improved understanding of the behavior of the complex subsystems in postural control.

## Conclusion

Frequency-specific Fractal Analysis has opened the way to systematic frequency-specific scaling markers which can be useful in many fields. We have shown here in an exploratory study that postural control strategies can be better understood and quantified using frequency-specific scaling exponents.

## Ethics statement

This study was approved by the institutional board of the faculty, with written informed consent from all subjects.

## Author contributions

PG designed the method with a rather mathematical insight, led data computation and wrote the article. VD-A managed the exploratory study design and its good application. EB led and provided complementary data (not expressed in the article) which helped to design the method. LA participated in the design of the method from a rather physiological perspective and participated in the writing of the article.

### Conflict of interest statement

The authors declare that the research was conducted in the absence of any commercial or financial relationships that could be construed as a potential conflict of interest.
