# Corrigendum: Frequency-Specific Fractal Analysis of Postural Control Accounts for Control Strategies

**DOI:** 10.3389/fphys.2018.01566

**Published:** 2018-11-02

**Authors:** Pierre Gilfriche, Véronique Deschodt-Arsac, Estelle Blons, Laurent M. Arsac

**Affiliations:** ^1^CATIE - Centre Aquitain des Technologies de l'Information et Electroniques, Talence, France; ^2^Univ. Bordeaux, CNRS, Laboratoire IMS, UMR 5218, Talence, France

**Keywords:** postural control, center-of-pressure, fractal physiology, DFA, FsFA, 1/f scaling

In the original article, there was an error. In the Discussion, we analyze how the scaling exponents computed on the differentiated signal and the original signal can be used to describe the frequency content of the signal in a global model. When differentiating the signal, we supposed that the value of β increased by 2, however, as β is the opposite of the slope in the power spectral density, the value of β decreased by 2 when the signal is differentiated. Thus, the equations β_1_ = 2 × α_1_ +1 and β_1_ = (2 × α_1_−1)+2 are incorrect and should be replaced by: β_1_ = 2 × α_1_−3 and β_1_ = (2 × α_1_−1)−2.

A correction has been made to Discussion, Interpretations and confrontation with other theories, paragraph two:

Indeed, Figure 7 shows that the whole frequency content of the CoP velocity signal could then be described by four parameters:

- P_f0_ (or AAMV)
- β_1_, the opposite of the slope of the low-frequency antipersistent range, with
  $$\begin{array}{l} \beta_{1}=2\times \alpha_{1}-3 \end{array}$$
- α_1_ being here the frequency-specific fractal exponent associated with visuo-vestibular loops (low-frequencies) in CoP position. This result is obtained by the fact that β_1_ is computed from the CoP velocity while α_1_ is computed from CoP position, its integral, so more precisely: β_1_ = (2 × α_1_−1)−2.
- β_2_, the opposite of the slope of the high-frequency persistent range, with
  $$\begin{array}{l} \beta_{2}=2\times \alpha_{2}-1 \end{array}$$
- α_2_ being here the frequency-specific fractal exponent associated with proprioceptive loops (high-frequencies) in CoP velocity.
- f_0_, the crossover frequency, which might be variable from one individual to another but is likely between 0.5 and 2 Hz according to the literature.

The authors apologize for this error and state that this does not change the scientific conclusions of the article in any way. The original article has been updated.

## Conflict of interest statement

The authors declare that the research was conducted in the absence of any commercial or financial relationships that could be construed as a potential conflict of interest.

